# Biopsychosocial view to pseudocyesis: A narrative review

**Published:** 2017-09

**Authors:** Marzieh Azizi, Forouzan Elyasi

**Affiliations:** 1 *Research Student Committee, Nasibeh Nursing and Midwifery Faculty, Mazandaran University of Medical Science Sari, Iran.*; 2 *Department of Psychiatry, Psychiatry and Behavioral Sciences Research Center, Addiction Institute, School of Medicine, Mazandaran University of Medical Sciences, Sari, Iran.*

**Keywords:** Pseudocyesis, Neuroendocrinology, Psychiatric disorders, Biopsychosocial, Socio-cultural

## Abstract

**Background:**

Pseudocyesis is a psychopathological clinical syndrome in which a non-pregnant woman firmly believes herself to be pregnant and manifests many symptoms and signs of pregnancy. Although the exact etiology of pseudocyesis has not been determined.

**Objective:**

This study was conducted with the aim of assessing the biopsychosocial view to pseudocyesis.

**Materials and Methods:**

A comprehensive search in electronic databases such as Google Scholar, PubMed, ScienceDirect, Web of Science, and Scopus was conducted between 1943-2016 to retrieve pseudocyesis related articles. For this purpose, 1149 articles were collected and 66 items were used.

**Results:**

Findings were classified into 2 main categories: a) pseudocyesis etiology, which could include (biological psychological factors and psychiatric disorders, and social factors); and b) pseudocyesis management.

**Conclusion:**

Pseudocyesis results from a multidimensional group of factors, and a holistic and comprehensive approach should be taken to its treatment. Cooperation between gynecologists and psychiatrists would likely be useful in addressing the condition.

## Introduction

Pseudocyesis is a psychopathological clinical syndrome in which a non-pregnant woman firmly believes she is pregnant and manifests many of the symptoms and signs of pregnancy (1-3). Pseudocyesis is categorized in the Diagnostic and Statistical Manual of Mental Disorders Forth edition under other specified somatic symptom and related disorder specified (4, 5). The term pseudocyesis derives from the Greek words pseudo (false) and kyesis (pregnancy) (6, 7). 

In different contexts, some synonyms for pseudocyesis (a few of which are no longer in common use) have included pseudopregnancy, imaginary pregnancy, simulated pregnancy, phantom pregnancy, hysterical pregnancy, and spurious pregnancy (8-10). With pseudocyesis, physiological manifestations of pregnancy occur, including irregular menstruation (e.g., amenorrhea or hypo-menorrhea), abdominal distention, the subjective sensing of fetal movements, changes in breast size or shape, milk secretion, darkening of areolar tissue, galactorrhea, weight gain, nausea and vomiting, and changes in the uterus and cervix (6, 7, 11-13). Of these symptoms, menstrual disorders and changes in breast size or shape are the most common (6, 14-17). Symptom duration usually varies from a few weeks to 9 months (18, 19).

Reliable, specific information about the frequency of pseudocyesis has not been compiled. However, the studies have shown that its incidence was greater in the past than it is today, with incidence ranges are given in the U.S. at 1 case per 250 pregnancies in 1940, and 1 to 6 cases per 22,000 deliveries in 2007 (6, 10, 20-22). In Nigeria, the frequency of pseudocyesis was reported 1 in 344 pregnancies in one study (20, 22-24). Pseudocyesis has also been reported in 1 of 160 women who were treated for infertility in Sudan (10). 

In most reported cases, pseudocyesis occurred in infertile and perimenopausal women between the ages of 20 and 44 yrs; in 80% cases, women with the condition were married (19, 22, 23, 25). This syndrome is rarely observed in postmenopausal women or men, or during adolescence or childhood (25, 26). Pseudocyesis occurs in patients with certain types of organic cerebral or neuroendocrinological pathology, chronic psychiatric disorders, and undiagnosed psychological or organic disorders (25). Some studies have indicated that not all pseudocyesis cases are rooted in a history of psychological problems (25, 27). In the absence of an exact etiology, studies have shown that different factors, including neuroendocrine disorders, physiological disorders, socio-cultural factors, and psychological or psychiatric factors or conditions are involved in the development of pseudocyesis (10, 14, 15, 19, 23, 24).

A comprehensive search of available databases suggests that large-scale studies have not been performed on pseudocyesis due to the small number of available patients; most of the information on the context of pseudocyesis and its development has been drawn from case reports (28-30). Psychiatry textbooks also have limited information about pseudocyesis. 

The present study was conducted with the aim of assessing pseudocyesis using a holistic approach and also to determine the biopsychosocial view to pseudocyesis.

## Material and methods

The initial literature search was independently conducted by titles and abstracts screening and 1149 articles was obtained. Finally, 66 articles remained for study inclusion. This study takes the form of a narrative review conducted in 5 steps: 1) identification of the research question; 2) comprehensive literature search to find relevant articles; 3) study selection; 4) ethical considerations; 5) data extraction (31).

**Research question** **identification**

Given the lack of clarity concerning the etiology of pseudocyesis, this study was interested in the specific biopsychosocial view to pseudocyesis. Accordingly, the research question was defined as: What is the biopsychosocial view to pseudocyesis?

**Comprehensive literature search to find relevant articles**

Following databases has been searched for relevant articles: Google Scholar, PubMed, ScienceDirect, Web of Science, and Scopus. The following key search terms (as per the MeSH) were used to retrieve articles published between 1943-2016: [pseudocyesis OR pseudopregnancy OR false pregnancy OR couvade syndrome OR simulated pregnancy OR delusions of pregnancy] AND [neuroendocrine OR endocrinology OR hormonal changes] AND [biologic OR physiologic OR physiology OR biopsychosocial] AND [socio-cultural OR cultural OR socio-economic OR social] AND [psychological issue OR mental problems OR psychiatric disorders] AND [etiology]. 

**Study selection**

**Figure 1:**
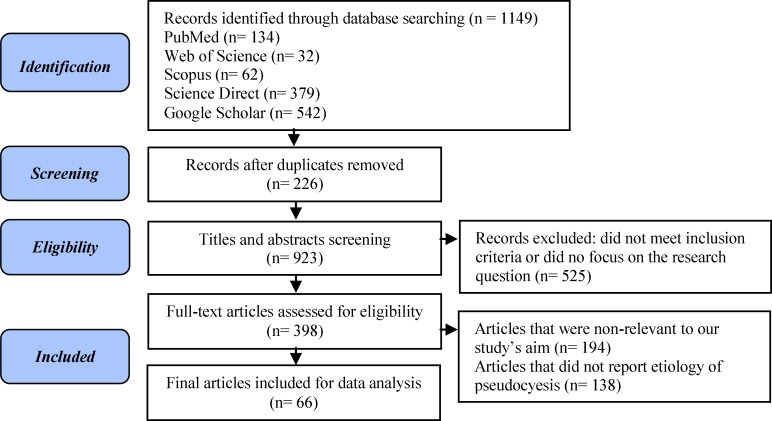
The process of search

Relevant articles have been chose according to the following inclusion criteria: published in scientific journals, and focused on pseudocyesis. After deleting repeated citations (n=226), 923 articles remained. During the abstract screening, 525 articles were excluded due to no focus on this study research question. Also during full-text review and appraisal, articles that did not consider different issues related to the etiology of pseudocyesis (n=138) or had aims other than this study’s aim (n=194) were excluded. Finally, 66 articles remained for study inclusion (Figure 1). 

**Ethical considerations**

Ethical considerations and the general standards for publication with respect to plagiarism, misconduct, data fabrication and/or falsification, double publication and/or submission, redundancy, and so forth have been completely adhered to by authors.

**Data extraction**

The full text of included articles was read carefully and relevant and required data for findings compilation were extracted and categorized.

## Results

The results of the literature review were classified into two main categories: a) etiology (including biological factors, psychological factors psychiatric disorders, and social factors), and b) management of pseudocyesis.

**Etiology**

As a multi-factorial disease, pseudocyesis is influenced in its development by several different elements, including neuroendocrine, social, psychodynamic, and cultural issues (Table I).

**Biological factors**

Different studies have proposed that biological factors play an important role in the etiology of pseudocyesis (15, 21, 32, 33). Neuroendocrinological changes or disturbances have been described as part of the onset of pseudocyesis (3, 10, 21, 33). Although most of these changes are not completely particular to pseudocyesis, abnormalities in the hypothalamic-pituitary-ovarian axis seem common in this condition’s pathology (15, 33, 34). In a few studies that examined the role of neuroendocrinological changes in pseudocyesis, the results showed that women with pseudocyesis may experience increased nervous system activity or dysfunction of the central nervous system (10, 21, 35).

A deficiency of dopamine is often observed in pseudocyesis; dopamine inhibits the gonadotropin-releasing hormone, luteinizing hormone (LH) and prolactin levels (13, 32, 36). Studies revealed that catecholamine and dopamine deficiency are responsible for hyperprolactinemia (8, 10, 37). Elevated prolactin levels lead to galactorrhea or lactation, the persistence of corpus luteum, and suppression of Follicle-stimulating hormone and LH secretion, ultimately resulting in amenorrhea (13, 14, 25, 29, 35, 36, 38). Corpus luteum is an initial source of circulating progesterone during the menstrual cycle, pregnancy, and pseudopregnancy (25, 39). Other neuroendocrinologic disturbances (e.g., decrease in LH and an excessive prolactin response to thyrotropin-releasing hormone stimulation, abnormality in growth hormone, Adrenocorticotropic hormone and cortisol levels were seen in pseudocyesis cases (15, 24). For disorders of the hypothalamic-pituitary-ovarian axis, psychiatric evaluation may be appropriate and valuable, with a focus on depression, anxiety, and marital problems along with psychiatric interventions as needed (40).

Treatment with psychotropic medications such as antipsychotics significantly induced lactation and amenorrhea, especially in women whose desire to have a child helped cause a belief that they were pregnant (25, 41, 42). The physiological events associated with pseudocyesis in the context of obstetric and gynecological issues include; recurrent abortions, the threat of menopause, and sterilization surgery (hysterectomy) (19). Numerous pathological conditions, including uterine or ovarian tumors, hydatidiform mole, ovarian cysts (13, 18, 43), uterine fibroids, morbid obesity or ascites, urinary retention, ectopic pregnancy, or central nervous system tumors, can also cause women to erroneously believe that they are pregnant (1, 10). With respect to psychiatric conditions, schizophrenia induces significant dopamine hypo-activity in different regions of the brain. Dopamine in the central nervous system increases the possibility of some neurochemical relationships between schizophrenia and pseudocyesis. Physicians should be considered iatrogenic factors in schizophrenic patients with pseudocyesis (25).

**Psychological factors and psychiatric disorders**

Various studies have noted the importance of fully accounting for psychological symptoms and overarching psychiatric disorders in those with pseudocyesis (3, 7, 15, 24).

**Psychological factors**

The relation between psychological issues and pseudocyesis is well recognized (28, 44). Ambivalence toward the existence of pregnancy or about wanting to be pregnant, the desire to have a child, fear of pregnancy, major psychological stress, hostile-aggressive attitudes regarding pregnancy and motherhood, challenges regarding gender identity or sexuality, a grief reaction following tubal ligation, or hysterectomy are some psychological issues that contribute to the etiology of pseudocyesis (2, 4, 14, 22, 25, 36, 45-50).

In psychologically mediated pseudocyesis, patients either avoid confronting or reject reality (the pregnancy is an illusion) and refuse gynecological assessment. Symptoms are accompanied by avoidance, minimization, and somatization (25). Case reports have reviewed patients with history of psychological factors that include severe childhood deprivation, disturbance of family vitality, significant separation anxiety and feelings of emptiness, a history of infertility, low frustration tolerance, inability to resolve tension, cognitive misinterpretation of bodily sensations and physical changes, emotional attachment (e.g., a strong bond between mother and daughter), childhood sexual abuse and emotional turmoil (6, 9, 10, 19, 44, 45, 49, 51-52). Several studies have also suggested that some kind of psychological loss can lead to pseudocyesis. These losses could include loss of love or loss of the object of one’s love; loss of reproductive capacity, loss of the child, and low self-esteem (6, 23, 45, 51). Psychologically, pseudocyesis has been considered as an illusion, a conversion, a delusion, and as a hysterical identification, yet specific links have not been proposed among these concepts (1, 7, 51).

**Psychiatric disorders**

Overall, the available psychiatric literature notes that pseudocyesis is mostly associated with disorders such as schizophrenia, anxiety disorders, mood disorders (including major depressive disorder, postpartum depression, bipolar disorder), affective disorder, conversion neurosis, and psychosis and personality disorders like dependent, histrionic, and borderline-personality disorders (14, 22, 24, 41, 55).

Review of the psychiatric literature suggests that depression has a crucial effect on the etiology of pseudocyesis. In some cases, when a patient understands that there is no pregnancy, she may react with a severe depressive episode (25). Most women with pseudocyesis suffer from mild to major depression, anxiety, or emotional stress due to psychological conflicts (3, 4 56). Depression can directly lead to obesity or weight gain in women due to sedentary behavior, unhealthy diet, and use of psychiatric medications, as the occurrence of amenorrhea, may cause women to believe that they to be pregnant (13).

The relationship between schizophrenia and pseudocyesis was assessed in a pair of studies (11, 24). Illusions and hallucinations, depersonalization, derealization, and personality transformation are the most common symptoms of pseudocyesis that manifest in schizophrenic patients (22). Pseudocyesis manifests differently depending on the psychiatric disorder present. Patients with pseudocyesis and manic depression have grandiose ideas and thoughts, while patients with depression often will have pseudocyesis as a postpartum psychosis or experience pseudo-hallucinations. In women suffering from anxiety disorders, prolonged stress with dissociative reactions may lead to pseudocyesis development (22). The sense of uterine contractions and fetal movements has been observed in manic and highly anxious patients (25). A study showed that the manifestation of pseudocyesis can be the first manifestation of psychosis (25). 

Pseudocyesis also occurs in patients without a history of psychopathology or personality disorder. These patients do not manifest fluctuations in cognitive level, disorientation, or memory loss. Personality disorders in patients with pseudocyesis include histrionic and borderline personality disorders and those with conflicting feelings regarding pregnancy (25).

**Social factors**

Although little information is available about the relationship between social factors and pseudocyesis, the available information supports the role of social factors in the development of pseudocyesis (19, 45, 57). Factors such as low educational attainment or lack of literacy, marital issues such as marital dissatisfaction, steady relationship or instability of relationship patterns, previous and current history of partner abuse, chronic social deprivation or social isolation, a background characterized by poverty and deprivation, family problems or poor family support, family and relative pressures to become pregnant, lower socioeconomic status, and unemployment are important in the manifestation of pseudocyesis but have not been explored and understood completely (4, 13-15, 19, 23, 29, 45, 50, 54, 58).

A study showed that pseudocyesis is more common in rural, undeveloped or underdeveloped countries where women never see physicians for an evaluation until they experience pain from labor. In contrast, women in developed countries often understand their false pregnancy (through pregnancy tests or ultrasonic examination) during the first trimester (10). Pseudocyesis is found in societies with rigid cultural and religious ideas, including those that place a high degree of social pressure on women to have more children and particularly more male children (4, 22, 24, 25). Studies in African countries have shown that the incidence of pseudocyesis is related to the importance of the fertility status of these women and is accounted of women values (19). In male-dominated societies (in which infertility usually results in divorce or a second marriage), infertile women use pseudocyesis as a defense mechanism to prevent these outcomes (12, 58).

**Management of pseudocyesis**

The effectiveness of treatment of pseudocyesis has not been measured via specific endpoints and outcomes in many studies. However, the published literature does provide a guide to what treatment strategies are useful and successful in addressing this disorder (1, 7, 16, 21, 29, 33, 50, 58-61). Although there is no accepted clinical protocol regarding management of women with pseudocyesis, successful treatment requires multidimensional cooperation between gynecologists, psychiatrists, and psychologists (15). Therapy might focus on helping the patient perceive the meaning of the symptoms and help resolve the stressors that were partly responsible for the condition’s onset (15). Obtaining a psychiatric history and clinical counseling should be considered as part of psychological management (17, 23, 49, 62). Physicians should communicate empathetically or have a good rapport with the patient; this will help immensely with proving the absence of pregnancy with pregnancy tests such as measurement of beta-chorionic gonadotropin (βCHG), thyroid gland hormones, and ultrasonic examination (13, 15, 23, 25). Sometimes, patients don’t accept the diagnosis and refer to different physicians to accept their claims (15). A study showed that pseudocyesis resolved in most patients who were confronted with the reality that they were not pregnant (25). 

Psychiatric procedures that can be used in these patients include supportive, cognitive, behavioral and psychoanalytical psychotherapy that focuses on problem-solving (13, 23, 58, 63). A combination of psychotherapy, pharmacotherapy with antidepressants or antipsychotics, hormonal therapy, and uterine curettage is effective in almost all or all patients (22, 25). Enlisting the help and support of family members and friends is vital (15, 50). In most cases, therapy will be accelerated by the patient’s interest in symptom resolution (49). Pseudocyesis may recur. Recovery from pseudocyesis is often spontaneous, but more often preceded by labor pains (20). 

**Table I:** Related bio-psychosocial factors of pseudocyesis

**Related biopsychosocial factors**		**Related papers (n)**
Biological factors		
	-Neuroendocrinological changes or disturbances AND Abnormalities in the HPO axis	11
	-Increased nervous system activity or dysfunction of the central nervous system (deficiency of dopamine)	3
	-Hormonal changes (inhibition of the gonadotropin-releasing hormone, LH and elevated prolactin levels, abnormality in GH, ACTH and cortisol levels).	18
	-Treatment with psychotropic medications such as antipsychotics	3
	-Obstetric and gynecological issues (recurrent abortions, threat of menopause, and sterilization surgery)	1
	-Pathologic medical conditions (uterine or ovarian tumors, hydatidiform mole, ovarian cysts, uterine fibroids, morbid obesity or ascites, urinary retention, ectopic pregnancy)	4
Psychological factors and psychiatric disorders		
	-Ambivalence toward the existence of pregnancy	2
	-The desire to have a child, fear of pregnancy, history of infertility	4
	-Major psychological stress, hostile-aggressive attitudes regarding pregnancy and motherhood	4
	-A grief reaction following tubal ligation, or hysterectomy	5
	-Severe childhood deprivation, disturbance of family vitality	2
	-Significant separation anxiety and feelings of emptiness, low frustration tolerance, inability to resolve tension	1
	-Cognitive misinterpretation of bodily sensations and physical changes	2
	-Childhood sexual abuse and emotional turmoil	5
	-Psychological losses (loss of love or loss of the object of one’s love); loss of reproductive capacity	4
	-Psychiatric disorders (major depressive disorder, postpartum depression, bipolar disorder), anxiety disorders, conversion neurosis, and psychosis and personality disorders	10
	-Schizophrenia	5
Social factors		
	-Low educational attainment or lack of literacy	3
	-Marital issues such as marital dissatisfaction, steady relationship or instability of relationship patterns	2
	- Previous and current history of partner abuse	3
	- Chronic social deprivation or social isolation	5
	-Family problems or poor family support, family and relative pressures to become pregnant	3
	-Lower socioeconomic status, unemployment	5
	-Rigid cultural and religious ideas	6

HPO: hypothalamic-pituitary-ovarian

LH: Luteinizing hormone

ACTH: Adrenocorticotropic hormone

GH: Growth hormone

## Discussion

The present study aimed to determine the biopsychosocial view to pseudocyesis, an abnormal state characterized by dysregulation of hormonal balance due to physical or psychological changes or a combination of these factors (33). This condition has been described in all nations, races, and social classes (18, 20). According to the psychiatric literatures, pseudocyesis has been categorized under various disorders, including hysteric conversion reaction or somatoform disorders (2, 21, 36, 50). In pseudocyesis, patients always strongly wish to have a child, but either prefer to avoid pregnancy or are infertile and therefore unable to become pregnant; the desire to have a child and the fear of the concept or state of being pregnant occur simultaneously (19, 25).

The incidence of pseudocyesis is different all over the world. These differences are related to the different values societies worldwide have concerning pregnancy and the role of women; differences in technological equipment for pregnancy testing; the availability of ultrasound for early diagnosis of pseudocyesis; and the different definitions of the condition (15). Results from one study indicated that the current tendency towards smaller family sizes in developed countries has resulted in a reduced incidence of pseudocyesis (13).

Pseudocyesis should be differentiated from delusion of pregnancy (appears as a symptom of a psychotic disorder and more commonly occurs in those with schizophrenia, schizoaffective disorder, delusional disorder, intellectual disability, elderly dementia, epilepsy, cerebral syphilis following encephalitis, and other organic brain syndromes), simulated pregnancy or malingering (person claims and admits to being pregnant despite his/her knowledge that she or he is not), Couvade syndrome (a phenomenon in which, during pregnancy, the husband of the pregnant woman manifests a variety of somatic and psychological symptoms such as anxiety, excitement, and agitation that closely simulate those of pregnancy) and pseudopregnancy (symptoms resembling pregnancy that are provoked by organic factors like endocrine changes caused by ovarian tumors) (25, 50, 64-66). The clinical differentiation between pseudocyesis and delusion of pregnancy is based on the presence or absence of the physical symptoms of pregnancy (45).

Despite the work that has taken place to research and medically assess the roots of pseudocyesis, the exact etiology of it is still unclear (19). Although this disorder may have a psychological basis, the process by which pseudocyesis develops is different in every patient (25). Most currently accepted theories focus on the interaction between psychological factors and reproductive problems, which are probably mediated by neuroendocrinological changes (19, 26). 

Some studies have considered pseudocyesis as a psychosomatic disorder (2, 25). Based on the results of case reports, pseudocyesis could also be considered a conversion reaction; major depressive disorder may also have an important role in etiology of pseudocyesis (18). Several hypotheses have been proposed along these lines (18). Three of these hypotheses are the psychosomatic, psychophysiological, and somatopsychic hypotheses. The classic psychosomatic hypothesis states that pseudocyesis begins with fantasies of pregnancy and leads to physiological symptoms (18, 21). The psychophysiological hypothesis showed that most patients with pseudocyesis have major depressive disorder or become depressed after resolution of the condition (21). The somatopsychic hypothesis asserts that pseudocyesis starts as random physiological changes and leads to the development of a pregnancy delusion in vulnerable individuals (18). The first studies on the disorder emphasized the physical characteristics of the disorder and its symptoms, and the etiology of pseudocyesis may be similar to that of delusions of pregnancy (1, 12). 

**Limitation**

This study limitation was that, we did not assess the quality of included articles. In this study, articles only in the English language were used and studies with other languages such as Persian and non-English studies even related to our study aim were ignored. 

## Conclusion

Given that pseudocyesis is rooted in multiple factors, a holistic and comprehensive treatment approach in this condition would appear to be most appropriate. Furthermore, due to the role of psychological and neuroendocrinological factors in the etiology of pseudocyesis, simultaneous treatment by a gynecologist and by psychiatrists can be effective and should lead to good clinical and therapeutic results. Given the mentioned limitations, this study researchers proposed that a systematic review or clinical trial regarding psychiatric aspects of pseudocyesis and psychotherapy in these patients will be required and useful.
